# Clinicopathologic Characteristics and Outcomes of Massive Multinodular Goiter: A Retrospective Cohort Study

**DOI:** 10.3389/fendo.2022.850235

**Published:** 2022-05-24

**Authors:** Qiang Chen, Anping Su, Xiuhe Zou, Feng Liu, Rixiang Gong, Jingqiang Zhu, Zhihui Li, Tao Wei

**Affiliations:** Department of Thyroid Surgery, West China Hospital, Sichuan University, Chengdu, China

**Keywords:** multinodular goiter, thyroidectomy, retrosternal extension, massive goiter, sternotomy

## Abstract

**Background:**

Thyroidectomy for massive goiters is challenging because of the increased risk of tracheomalacia, combined sternotomy, postoperative morbidity, and mortality, whereas studies investigating the clinicopathologic characteristics, postoperative morbidities, and surgical outcomes of massive goiters are limited.

**Methods:**

Patients with goiters undergoing thyroid surgery between 2009 and 2019 were retrospectively reviewed. A total of 227 patients were enrolled and divided into massive goiter group and large goiter group according to the weight of the goiter. Clinicopathologic characteristics, postoperative morbidities, and surgical outcomes were compared between the two groups.

**Results:**

Seventy-four patients (32.6%) had a goiter weighing more than 250 g and 153 patients (67.4%) were categorized in the large goiter group. Compared to large goiter patients, massive goiter patients had higher rates of retrosternal extension (82.4% vs. 30.7%), combined sternotomy (12.2% vs. 1.3%), intensive care unit admission (25.7% vs. 7.2%), transient hypoparathyroidism (41.9% vs. 25.5%), and transient recurrent laryngeal nerve palsy (10.8% vs. 3.3%) as well as prolonged length of hospital stay (*P* < 0.05).

**Conclusions:**

Massive goiter patients were at increased risk of combined sternotomy, intensive care unit admission, postoperative morbidities as well as prolonged length of hospital stay after thyroidectomy compared to large goiter patients, but most of them can be treated through a cervical approach with a favorable outcome.

## Introduction

Multinodular goiter (MNG) is a common thyroid disorder, which is estimated to affect about 1.5 billion people worldwide (1, 2). Iodine deficiency is considered to be the most common cause of goiter in iodine-deficient areas, and the incidence of nodular goiter has declined with the popularization and implementation of iodized salt intake in the last decades (3, 4). However, the prevalence of nodular goiter, in iodine repletion countries, is reported in a range from 13% to 45% (5). Most nodular goiters are generally asymptomatic and slow growing, while surgical intervention should be considered for patients with compressive symptoms, disfigurement, or suspected malignancy. The severity of compressive symptoms depends on the size and location of the goiter, which may worsen owing to the rapid growth or hemorrhage. In a recent study (6), shortness of breath was noted in 40% of patients with large cervical goiter (>100 g) and 52% with substernal goiter.

Surgical intervention is the most effective treatment for massive goiters that offers a definitive effect on alleviation of compressive symptoms. However, patients with massive goiters pose airway and surgical challenges due to the airway deformity and distorted neck anatomy. In addition, retrosternal extension, with a reported incidence of 12%~46%, is another challenge to surgeons because of the risk of an extra-thoracic approach (7, 8). Therefore, it is important to conduct a detailed preoperative evaluation as well as peri-operative management in patients with massive goiters. Although there have been many studies focusing on large goiters (>100 g), there are few studies targeting massive goiters.

The aim of this study was to investigate the clinicopathologic characteristics, postoperative morbidities, and surgical outcomes of patients with massive goiters.

## Materials and Methods

### Study Design

We conducted a retrospective study of patients who underwent thyroidectomy as treatment for goiter in West China Hospital, Sichuan University from September 2009 to December 2019. Clinical and pathological data were retrieved from electronic medical record system from the Computer Information Resource Center of West China Hospital. Demographic and clinicopathologic variables, including age, gender, compressive symptoms, retrosternal extension, surgical procedures, postoperative morbidities, length of hospital stay, concurrent thyroid malignancy, and surgical outcomes, were analyzed and compared between the large goiter group and the massive goiter group. Patients with incomplete record, in absence of preoperative CT record, or in absence of goiter weight record as well as an ectopic goiter were excluded. This study was designed according to the STROBE criteria and was approved by the Institutional Review Board of West China Hospital, Sichuan University.

### Peri-Operative Management

All patients underwent a preoperative thyroid function test including thyroid stimulating hormone, free triiodothyronine, free thyroxine, thyroglobulin, and antibodies against thyroperoxidase and thyroglobulin as well as serum parathyroid hormone (PTH). Patients with hyperthyroidism in the study were treated with methimazole (10-30 mg/day; Merck KGaA, Darmstadt, Germany) to achieve a euthyroid state prior to surgery. Both ultrasonography (US) and cervicothoracic CT scan were performed in all patients to assess the size, location, and adjacent structures of the goiter. A fiberoptic laryngoscopy was performed preoperatively to assess vocal cord mobility. Post-operative laryngoscopy was selectively performed in patients with voice change. Fine-needle aspiration cytology (FNAC) was selectively performed in patients with suspected malignancy based on preoperative US findings. Moreover, an esophageal or tracheal endoscopy was performed in patients with suspected malignancy or significant symptoms of dysphagia and dyspnea.

Serum calcium and PTH levels were routinely assessed for all patients on the first day after surgery. Intravenous and oral calcium supplementations with 1,25-dihydroxyvitamin D3 (1.8~2.4 g/day; Caltrate, Wyeth, New Jersey, USA) were prescribed if the patient exhibited symptoms of hypocalcemia. The dosage of these medications was gradually tapered off with the normalization of serum calcium and PTH levels.

### Definitions

Although there is no uniform definition of a large goiter, several studies defined a large goiter as the gross weight of more than 100 g (6, 7). Therefore, a large goiter was also defined as a goiter with a gross weight > 100 g, and a massive goiter was defined as a goiter with a gross weight >250 g in the present study. Similarly, a substernal goiter was defined as a goiter extending below the plane of the thoracic inlet on computed tomography (CT) scan in the supine position.

Hypoparathyroidism was defined as a serum PTH level lower than 1.6 pmol/L with a concurrent serum calcium level <2.0 mmol/L. Permanent hypoparathyroidism was defined as postoperative hypocalcemia for more than 6 months. Vocal cord palsy was diagnosed if vocal cord immobility was confirmed by laryngoscopy. Permanent recurrent laryngeal nerve (RLN) palsy was defined as persistent vocal cord palsy for more than 12 months postoperatively.

### Statistical Analysis

Normally or nonnormally distributed continuous variables were described as mean ± standard deviation (SD) or median, respectively. Comparisons between two groups were conducted using Pearson’s chi-square test or Fisher’s exact test for categorical variables, whereas continuous variables were compared using Student’s *t*-test or Mann-Whitney *U* test. Statistically significant was accepted when *P* < 0.05. All statistical analyses were performed using SPSS Statistics software version 21.0 (IBM Inc., Chicago, IL, USA).

## Results

### Patient Characteristics

**Figure 1:**
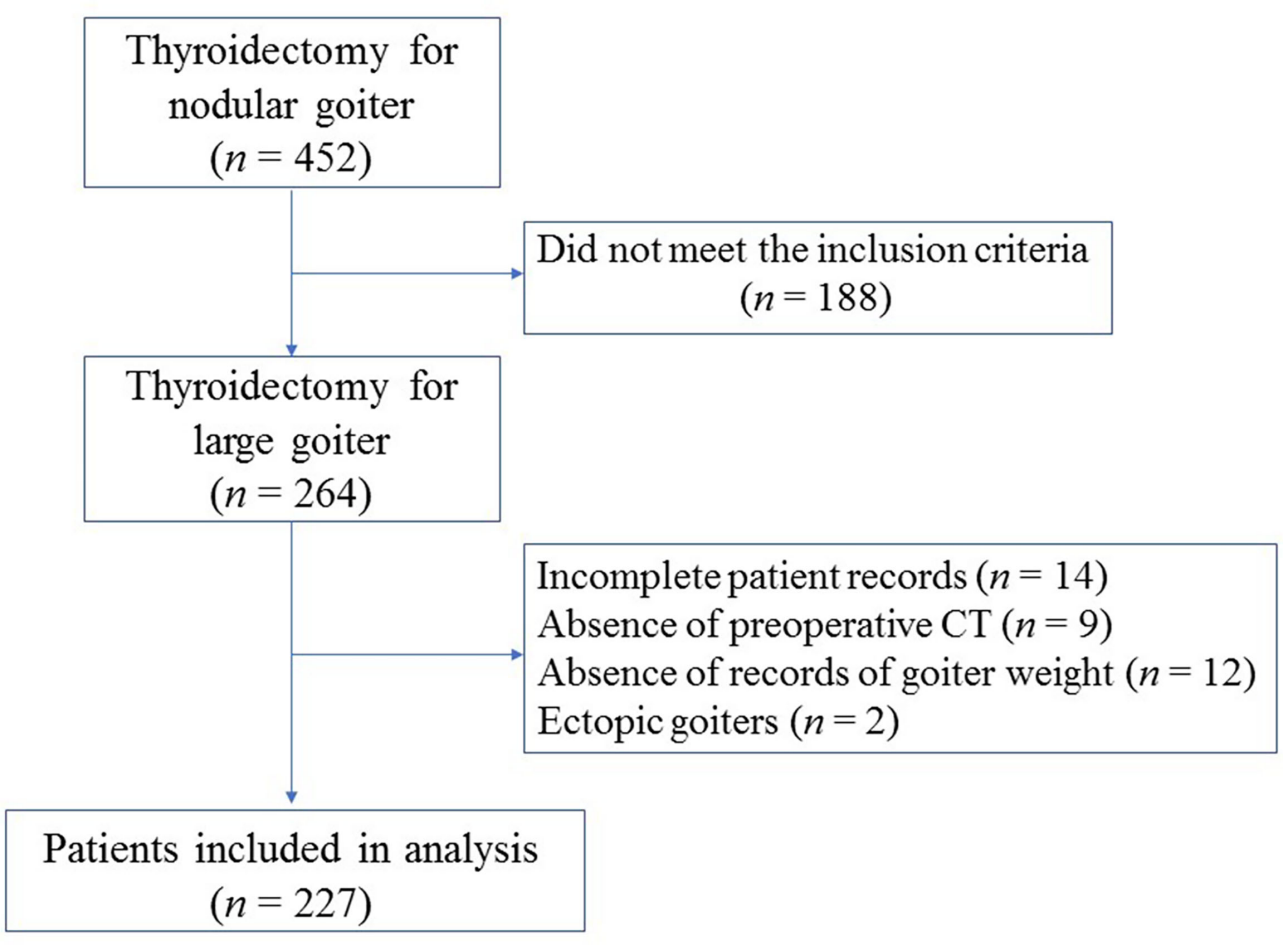
Flow chart of patients’ inclusion.

From September 2009 to December 2019, a total of 452 thyroidectomies were performed for nodular goiters in our department. Finally, a total of 227 patients who met the criteria were included (**Figure 1**). Patient demographics and characteristics are summarized in **Table 1**. There were 171 females and 56 males, with a median age of 58 years (range, 17-87 years). The most common compressive symptom was dyspnea (44.9%), followed by dysphagia (21.6%). Of these, 159 patients (70.0%) had a normal thyroid function, while 21 patients (9.3%) exhibited hyperthyroidism and 47 (20.7%) exhibited hypothyroidism. Total thyroidectomy or near total thyroidectomy was performed in 93 patients (41.0%), lobectomy in 62 patients (27.3%), Dunhill’s operation in 29 patients (12.8%), and 43 patients (18.9%) underwent completion thyroidectomy. In addition, concurrent central lymph node dissection (level VI) was performed in 21 patients (9.3%).

**Table 1:** Demographic and clinical characteristics of patients.

	n = 227
Age at presentation (y), median (range)	58 (17-87)
Gross thyroid weight (g), mean ± SD	284.2 ± 54.6
Gender, *n* (%)	
Male	56 (24.7)
Female	171 (75.3)
Preoperative thyroid function, *n* (%)	
Euthyroidism	159 (70.0)
Hyperthyroidism	21 (9.3)
Hypothyroidism^*^	47 (20.7)
Hashimoto thyroiditis, *n* (%)	35 (15.4)
Preoperative symptoms, *n* (%)	
Dyspnea	102 (44.9)
Dysphagia	49 (21.6)
Hoarseness	13 (5.7)
Neck pain	35 (15.4)
Retrosternal extension, *n* (%)	108 (47.6)
Surgical procedure, *n* (%)	
Lobectomy	62 (27.3)
Dunhill’s operation^#^	29 (12.8)
Total or near total thyroidectomy	93 (41.0)
Completion thyroidectomy	43 (18.9)
Central lymph node dissection	21 (9.3)
Sternotomy	11 (4.8)
Pathology, *n* (%)	
Benign	204 (89.9)
Malignant	23 (10.1)
Length of hospital stay (d), mean ± SD	8.7 ± 2.6

SD, standard deviation.

*including subclinical hypothyroidism.

^#^Dunhill’s operation: one side lobectomy and one side subtotal thyroidectomy.

Retrosternal extension was observed in 108 patients (47.6%). Based on careful review of CT images, the inferior limit of goiter extended over the aortic arch in 9 patients (4.0%) and to the tracheal bifurcation in 2 patients (0.8%). Malignancy was identified in 23 patients (10.1%) and the type of malignancies included papillary microcarcinoma (n = 13), papillary carcinoma (n = 8), and follicular papillary carcinoma (n = 2). Among them, 16 cases were diagnosed by FNAC, while other 7 cases with negative FNAC results were confirmed by intraoperative frozen section examination or by paraffin pathological diagnosis.

The mean ± SD weight of goiter was 284.2 ± 54.6 g. One hundred and fifty-three patients (67.4%) were categorized in the large goiter group and 74 patients (32.6%) had a goiter weight >250 g. As shown in **Table 2**, massive goiter patients were more frequently from rural areas (*P* = 0.032) with a longer duration course of goiter (*P* < 0.001) compared to large goiter patients. The rates of compressive symptoms (*P* < 0.05) and retrosternal extension (*P* < 0.001) were significantly higher in the massive goiter group. Moreover, patients with massive goiters more frequently had a previous thyroidectomy (*P* < 0.001) with significantly higher rates of hypothyroidism (*P* = 0.002), combined sternotomy (*P* < 0.001), and prolonged length of hospital stay (*P* < 0.001).

**Table 2:** Age and gender demographics of the double Sinopharm Vaccinated cohort in this study.

	Large goiters (≤250 g) (*n* = 153)	Massive goiters (>250 g) (*n* = 74)	P
Age at presentation (y), median (range)	54 (17-72)	63 (26-87)	0.526
Gender, *n* (%)			0.119
Male	46 (30.1)	15 (20.3)	
Female	107 (69.9)	59 (79.7)	
BMI (kg/m^2^)			0.408
<25	74(36)	40(4)	
≥25	43	30	
Duration of goiter (y), mean ± SD	4.3 ± 2.3	10.5 ± 5.2	<0.001
Resident region, *n* (%)			0.032
Urban	66 (43.1)	21 (28.4)	
Rural	87 (56.9)	53 (71.6)	
Hypothyroidism*, *n* (%)	23 (15.0)	24 (32.4)	0.002
History of thyroidectomy^§^, *n* (%)	19 (12.4)	24 (32.4)	<0.001
Hashimoto thyroiditis, *n* (%)	23 (15.0)	9 (16.2)	0.817
Retrosternal extension, *n* (%)	47 (30.7)	61 (82.4)	<0.001
Dyspnea	41 (26.8)	61 (82.4)	<0.001
Dysphagia	24 (15.7)	25 (33.8)	0.002
Hoarseness	5 (3.3)	8 (10.8)	0.032^†^
Sternotomy, *n* (%)	2 (1.3)	9 (12.2)	<0.001^†^
Postoperative pathology, *n* (%)			0.002
Benign	144 (94.1)	60 (81.1)	
Malignant	9 (5.9)	14 (18.9)	
Length of hospital stay (d), mean ± SD	7.9 ± 2.4	9.8 ± 2.5	<0.001

SD, standard deviation.

*Including subclinical hypothyroidism.

^§^Including lobectomy and subtotal thyroidectomy.

^†^Fisher’s exact test.

### Primary Outcomes

Postoperative outcomes are shown in **Table 3**. One case of permanent hypoparathyroidism was recorded in the massive goiter group. There was a higher incidence of transient hypoparathyroidism in the massive goiter group compared to the large goiter group (41.9% vs. 25.5%, *P* = 0.012). Moreover, there was an increased risk of transient RLN palsy in the massive group (*P* = 0.032). No statistical differences were observed in permanent RLN palsy and permanent hypoparathyroidism between the two groups. Additionally, massive goiter patients had a significantly higher rate of intensive care unit (ICU) admission (*P* < 0.001). None of the patients in both groups required tracheostomy, while 5 massive goiter patients (6.7%) required prolonged intubation to prevent tracheomalacia, all of which were successfully extubated a few days later. Despite the higher rate of concurrent malignancy in the massive group, neither the patients in the large group nor in the massive group suffered recurrent disease with a mean follow-up time of 6.7 months (range, 3–18 months) and 14.2 months (range, 9–24 months), respectively.

**Table 3:** Comparison of postoperative complications between large goiters and massive goiters.

	Large goiters	Massive goiters	*P*
	(≤250 g) (*n* = 153)	(>250 g) (*n* =74)	
Transient hypoparathyroidism, *n* (%)	39 (25.5)	31 (41.9)	0.012
Permanent hypoparathyroidism, *n* (%)	0	1 (1.4)	0.326*
Transient RLN palsy, *n* (%)	5 (3.3)	8 (10.8)	0.032*
Permanent RLN palsy, *n* (%)	1 (0.7)	3 (4.1)	0.103*
Surgical reintervention for bleeding, *n* (%)	2 (1.3)	4 (5.4)	0.090*
Wound infection, *n* (%)	5 (3.3)	3 (4.1)	0.718*
ICU admission, *n* (%)	11 (7.2)	19 (25.7)	<0.001

RLN, recurrent laryngeal nerve; ICU, intensive care unit.

*Fisher’s exact test.

## Discussion

Goiter is defined as an enlargement of the thyroid gland that usually presents as a swelling in the front of the neck, which can be further classified as endemic or non-endemic, diffuse or nodular, and toxic or nontoxic (9). Most goiters are asymptomatic and do not require surgical intervention, while surgery is recommended in patients with compressive symptoms, suspected malignancy, drug-resistant hyperthyroidism, or retrosternal extension (10–12). Patients with large goiters frequently present with shortness of breath, dysphagia, and voice change; however, the most common symptom is nonspecific. In our study, the most common symptom was dyspnea, and patients in the massive goiter group had a much higher rate of compressive symptoms (for example, dyspnea, dysphagia, and hoarseness). Compressive symptoms may be directly related to goiter size, while goiter size is correlated with growth time: the longer the growth time, the larger the size. Consistent with the data of Agarwal et al. (13), we found that the duration course of goiter in the massive goiter group was longer than that in the large goiter group. These results, in a word, justify that a massive goiter potentially results from an untreated goiter with further progression for a long time.

Goiter can affect the thyroid gland diffusely or only involve one lobe. Although performing total thyroidectomy or lobectomy remains controversial, lobectomy may be an appropriate procedure for goiters which are isolated to one lobe. However, in a prospective study with a mean follow-up time of 14.5 years, Beatriz et al. (14) found that about 15% of patients (394/2,675) with MNG required completion thyroidectomy for recurrence. In this study, 43 patients (18.9%) underwent completion thyroidectomy; however, we could not conclude that the patients had recurrence of MNG because it is difficult to evaluate whether residual goiter remained after the initial surgery. Moreover, whether nodular disease involves the contralateral lobe is difficult to assess. Interestingly, we found that a higher proportion of patients with massive goiter had a previous thyroidectomy. In general, the residual thyroid tissue after lobectomy can secrete sufficient thyroid hormone to maintain daily metabolism, while thyroxine will be insufficient if the remnant of thyroid tissue is small or involved in diseases, such as Hashimoto thyroiditis or nodular goiter. Low-level of thyroid hormone stimulates the synthesis and secretion of TSH; the latter one plays an important role in the development of nodular thyroid hyperplasia (15, 16). As shown in the present study, the patients with massive goiter were found to have a higher rate of hypothyroidism, which suggests that close surveillance of thyroid function and appropriate supplementation of thyroxine may play an important role in preventing goiter progression.

Retrosternal extension, namely substernal goiter, is frequently associated with a large goiter. Although numerous definitions have been proposed and compared, there is no consensus on the definition of substernal goiter. In the literature, the reported incidence of substernal goiter varies from 12% to 46% due to the use of different criteria (7, 8). In the present study, 108 patients (47.6%) were confirmed with retrosternal extension, and all of them located in the anterior mediastinum. Moreover, we identified that the rate of retrosternal extension in the massive group was higher than that in the large group, which is consistent with a previous report (12). Most substernal goiters can be managed through a cervical approach; sternotomy should be taken into consideration if the extension is beyond the level of the aortic arch, in combination with malignancy, or in the presence of a posterior mediastinal goiter, ectopic goiter, or recurrent goiter (17–19). Only 11 patients (4.8%) in the study required a combined sternotomy, and all the patients who underwent a combined sternotomy had a goiter extending below the level of the aortic arch. Similar to the report by Sancho et al. (20), in which a goiter weight of over 250 g is an independent predictor of an increased need for additional sternotomy, suggesting that a much higher rate of sternotomy would be performed in patients with massive goiters. Cervical and thoracic CT scans play a valuable role in evaluating the depth and extent of substernal goiter and its relationship with surrounding structures, which is helpful in planning the surgical strategy and anesthesia intubation.

Total thyroidectomy for massive goiter is challenging, which is associated with a significantly higher risk of RLN and parathyroid damage (21, 22). Our data showed that the rate of RLN injury was 11.2%, which was comparable between the two groups. Lin et al. (23) reported that the incidence of right-sided RLN injury was more common than that of left-sided RLN injury. However, no such difference was noted between right-sided and left-sided RLN injuries in this study. In our experience, adequate exposure is crucial in ensuring safe thyroidectomy and preventing the potential risk of RLN injury. Recently, continuous intraoperative nerve monitoring has been reported as a useful and effective modality in identifying and preserving the integrity of the nerve, which effectively lower the incidence of postoperative vocal cord palsy (24). Postoperative hypoparathyroidism is not uncommon in large goiters due to an increased risk of inadvertent dissection or devascularization of parathyroid glands. The incidence of transient hypoparathyroidism was significantly higher in the massive goiter group, whereas the rate of permanent hypoparathyroidism was similar between the two groups. This may have resulted from the reason that patients undergoing lobectomy, Dunhill’s procedure, and completion thyroidectomy were also enrolled in the present study.

The incidence of malignancy in MNG has been reported to vary from 4% to 17% (25). In our study, the overall incidence of malignancy was 10.1% (23/227), and it was 18.9% (14/74) in the massive goiter group, which was significantly higher than that in the large goiter group. Whether there is an increased risk of malignancy in large goiter remains controversial; however, malignancy implies an increased risk of RLN and parathyroid injury due to the necessity of lymph node dissection. Although the relationship between goiter size and malignancy has not been clearly determined, it has been reported that malignancy was significantly higher in intrathoracic goiter (26, 27). On the basis of the study, a detailed preoperative US evaluation with FNAC is indispensable for massive goiters, even though the diagnostic accuracy of malignancy was relatively low in our study (69.6%). This can be attributed to the limitations of sonographic detection of surrounding structures, the deep location of the tumor, and/or tumor size <1.0 cm. In our experience, intraoperative frozen section examination provides a supplementary modality for the diagnosis of malignant nodules.

Some limitations should be noted in our study. First, retrospective analysis in general is subject to informational biases: data accuracy is closely dependent on the registration/codification process. Second, the present study was conducted in a single institution with a limited sample size. Finally, the rate of retrosternal extension was not accurately assessed and compared because there is no uniform definition of a retrosternal goiter. However, in our opinion, we prefer to define a substernal goiter that extending below the plane of the thoracic inlet on the basis of that researchers in different facilities and circumstances can easily and uniformly use this anatomic definition.

## Conclusions

Massive goiter (>250 g) patients were at higher risk of combined sternotomy, ICU admission, postoperative morbidities as well as prolonged length of hospital stay after thyroidectomy compared to large goiter patients. Although massive goiters were associated with an increased rate of retrosternal extension, most of them can be removed through a cervical approach. The overall surgical outcomes for massive goiters may potential less favorable than for large goiters, whereas most massive goiters can be safely excised with minimal morbidity with an experienced surgeon and a dedicated planning.

## Data Availability Statement

The original contributions presented in the study are included in the article/supplementary material. Further inquiries can be directed to the corresponding author.

## Ethics Statement

The study was approved by the Institutional Review Board of West China Hospital, Sichuan University. Written informed consent to participate in this study was provided by the patients or their legal guardian.

## Author Contributions

QC, data collection, analysis, drafting of manuscript, interpretation of data, final approval, and accountability for all aspects of the work. AS, data collection, interpretation of data, and final approval. XZ, interpretation of data, analysis, and final approval. FL and RG, data collection, analysis, and final approval. JZ, interpretation of data, critical review, revising, and final approval. ZL, interpretation of data, critical review, and final approval. TW, study design, interpretation of data, critical review, revising, final approval, and accountability for all aspects of the work. All authors contributed to the article and approved the submitted version.

## Funding

This study did not receive any specific grant from funding agencies in the public, commercial, or not-for-profit sectors.

## Conflict of Interest

The authors declare that the research was conducted in the absence of any commercial or financial relationships that could be construed as a potential conflict of interest.

## Publisher’s Note

All claims expressed in this article are solely those of the authors and do not necessarily represent those of their affiliated organizations, or those of the publisher, the editors and the reviewers. Any product that may be evaluated in this article, or claim that may be made by its manufacturer, is not guaranteed or endorsed by the publisher.
